# Mapathons versus automated feature extraction: a comparative analysis for strengthening immunization microplanning

**DOI:** 10.1186/s12942-021-00277-x

**Published:** 2021-06-07

**Authors:** Amalia Mendes, Tess Palmer, Andrew Berens, Julie Espey, Rhiannan Price, Apoorva Mallya, Sidney Brown, Maureen Martinez, Noha Farag, Brian Kaplan

**Affiliations:** 1 Division of Toxicology and Human Health Sciences, Agency for Toxic Substance and Disease Registry, 4770 Buford Hwy NE, Atlanta, GA 30341 USA; 2 Sustainable Development Practice, Maxar Technologies, 1300 W 120th Avenue, Westminster, CO 80234 USA; 3 Polio Program, Bill & Melinda Gates Foundation, 500 5th Ave N, Seattle, WA 98109 USA; 4 Global Immunization Division, Centers for Disease Control and Prevention, 1600 Clifton Rd, Atlanta, GA 30333 USA

**Keywords:** Feature extraction, Mapathon, Essential immunization, Population estimates, Microplanning, Satellite imagery, Building footprints

## Abstract

**Background:**

Social instability and logistical factors like the displacement of vulnerable populations, the difficulty of accessing these populations, and the lack of geographic information for hard-to-reach areas continue to serve as barriers to global essential immunizations (EI). Microplanning, a population-based, healthcare intervention planning method has begun to leverage geographic information system (GIS) technology and geospatial methods to improve the remote identification and mapping of vulnerable populations to ensure inclusion in outreach and immunization services, when feasible. We compare two methods of accomplishing a remote inventory of building locations to assess their accuracy and similarity to currently employed microplan line-lists in the study area.

**Methods:**

The outputs of a crowd-sourced digitization effort, or mapathon, were compared to those of a machine-learning algorithm for digitization, referred to as automatic feature extraction (AFE). The following accuracy assessments were employed to determine the performance of each feature generation method: (1) an agreement analysis of the two methods assessed the occurrence of matches across the two outputs, where agreements were labeled as “befriended” and disagreements as “lonely”; (2) true and false positive percentages of each method were calculated in comparison to satellite imagery; (3) counts of features generated from both the mapathon and AFE were statistically compared to the number of features listed in the microplan line-list for the study area; and (4) population estimates for both feature generation method were determined for every structure identified assuming a total of three households per compound, with each household averaging two adults and 5 children.

**Results:**

The mapathon and AFE outputs detected 92,713 and 53,150 features, respectively. A higher proportion (30%) of AFE features were befriended compared with befriended mapathon points (28%). The AFE had a higher true positive rate (90.5%) of identifying structures than the mapathon (84.5%). The difference in the average number of features identified per area between the microplan and mapathon points was larger (t = 3.56) than the microplan and AFE (t = − 2.09) (alpha = 0.05).

**Conclusions:**

Our findings indicate AFE outputs had higher agreement (i.e., befriended), slightly higher likelihood of correctly identifying a structure, and were more similar to the local microplan line-lists than the mapathon outputs. These findings suggest AFE may be more accurate for identifying structures in high-resolution satellite imagery than mapathons. However, they both had their advantages and the ideal method would utilize both methods in tandem.

## Background

Of the 20 million children across the world with incomplete or no essential immunization (EI) for vaccine-preventable diseases, nearly half live in countries with conflicts and population displacement (e.g., Afghanistan, Central African Republic, Iraq, Mali, Nigeria, Pakistan, and Somalia) [1]. Conflicts and regional instabilities generally lead to poor vaccination coverage and interrupted vaccine schedules [2] due to disruption of health systems and impeded access to care resulting in vaccine delivery inequities. Currently, the barriers to vaccine preventable disease control are less about pathogen biology and more about the identification of sub-populations missed by the Expanded Programme on Immunization and therefore left without equitable access to interventions like essential immunization and supplementary vaccination campaigns [3, 4]. Immunization programs miss or underserve hard-to-reach sub-populations for various reasons including geographic inaccessibility, irregular population migration due to regional instabilities, and nomadic lifestyles. For this reason, it remains imperative to employ innovative and effective technologies to improve remote identification of hard-to-reach sub-populations, thereby allowing service delivery during periods of accessibility.

Understanding the geographic distribution of target populations for health interventions is a critical component of microplanning—an epidemiologic database aimed at delivering health-care interventions like childhood essential immunizations by addressing the implementation demands of a specific setting [5]. Microplans critically inform decisions regarding appropriate delivery strategies (i.e., fixed-post, outreach, or mobile) and logistics needed to reach children targeted for the intervention (i.e., target populations) [6]. Each microplan is composed of a line-list where every row represents data pertaining to the geographic unit of analysis being studied while columns illustrate variables containing demographic information (example- children under 5 years of age, number of households to be visited, estimates of total resources needed, etc.). Despite the utility of current microplans, arguments have been made for updated methods of microplanning that leverage Geographic Information Systems (GIS) and satellite imagery to generate high quality and up-to-date maps of target population distributions and maps of built features such as residential structures and settlements [7, 8]. In their Reach Every District (RED) strategy for essential immunization, the World Health Organization (WHO) and the United Nations Children’s Fund (UNICEF) recognized the need for these updated methods and outlined new GIS-enhanced microplanning tactics for improved location surveillance of some populations.

In some situations, GIS-based microplanning incurs higher costs than traditional, non-GIS based microplanning; however, this does not necessarily imply cost ineffectiveness. A recent cost-effectiveness analysis conducted in two Nigerian states determined that increased cost for GIS-based microplanning was mostly due to purchasing additional vaccines for populations previously uncounted and unreached by traditional microplanning methods [7]. Not only does GIS-based microplanning save resources when executed appropriately, it also protects the lives of field workers in settings where conflict could compromise their security by reducing the need for deployment to high-risk areas [6]. When in-person access is safe and feasible, having field workers physically present in the region of interest allows for ground-truthing which is needed to validate maps generated remotely (i.e., generated using imagery and without physical access to the area of interest). Supplementing microplanning methods with the integration of GIS technologies could further support other public health interventions, such as spraying insecticides for mosquito abatement and malaria prevention [9, 10] and the provision of maternal and child health care services [7].

To support the integration of GIS technology in public health planning, researchers take advantage of high- or very high-resolution (VHR) satellite imagery generated by satellites like GeoEye, QuickBird, RapidEye, and WorldView. Sub-meter resolution imagery from these satellites allows analysts to digitize features such as buildings, rooftops, roads, nomadic camps, and informal settlements. The size of a population can even be modeled from these footprints.

Large-scale feature digitization (e.g., digitization of individual structures across multiple districts or provinces) from imagery without automated methods is very time-consuming for a small group of analysts, especially when the features of interest are sparse in the imagery. Consequently, a method of participatory data acquisition has gained popularity—the “mapathon”—which is a time-limited, crowd-sourced effort by a group of trained participants with or without formal geospatial analysis backgrounds. Participants, used in this paper to describe both the group of contributors and validators, generate spatial data of features like residential structures or informal settlements within a specific area of interest by using GIS platforms, such as OpenStreetMap and ArcGIS Online. Generally there is no financial incentive for contributions made during a mapathon [11] and anyone with a computer and internet connection can contribute. Consequently, humanitarian efforts frequently rely on mapathons to identify mobile populations and undetected settlements [11, 12]. Similarly, data generated from mapathons are useful for detecting and enumerating populations missed during immunization campaigns; thereby, optimizing immunization campaign microplans. Mapathons also provide data that are used to map health facility catchment areas when merged with other key information [12].

An alternative method to using mapathons is automated feature extraction (AFE), a type of model-based feature generation, which can be semi- (i.e., some human support) or fully automated (i.e., no human support). After an initial time investment to manually develop training data using selected examples of features of interest (e.g., man-made structures) and examples of features not of interest (e.g., large boulders), AFE does not require time-consuming and labor-intensive steps such as identifying structures and placing points or drawing polygons manually on a computer. AFE relies on computer algorithms and models to learn patterns, edges, and shapes of features (e.g., rooftops or settlement footprints) to digitize and categorize. Machine learning algorithms are designed to enhance performance by effectively teaching the computer how to extract the desired spatial data from imagery with both precision and accuracy. AFE has been leveraged for a myriad of purposes, such as mapping agricultural land use [13–16] and water boundaries [17, 18], estimating human and livestock populations [19, 20], road feature extraction [21, 22], building feature extraction [23–29], and to support disaster relief efforts [30, 31].

Like mapathons, AFE relies on high-resolution imagery for optimal performance, but image collection parameters can be refined to account for cloud cover, thick vegetation, and low spectral resolution. Additionally, using a time-series of images can improve the accuracy of feature detection by minimizing false-positives [14, 18] and is especially helpful when analyzing pre- and post-disaster impacts to roads [30] and facilities [31].

There is currently no information on how results from participatory mapping compare to the results from AFE; if researchers determine AFE to be as accurate and precise as mapathons but faster at generating spatial data, increasing its use could save valuable resources and time for public health programs without compromising quality. Additionally, as geospatial professionals gain a deeper understanding of the strengths of each method, future projects can more optimally combine the two to complement and enhance their end-products.

Disparities in equitable access to health services will decrease when additional sub-populations are identified in microplans and serviced by EI campaigns and other public health interventions. Here, we seek to explore and compare the accuracy of two methods of feature generation—mapathons and AFE—to provide evidence for the suitability of each method in identifying hard-to-reach populations vulnerable to vaccine-preventable diseases in inaccessible areas and whether the two methods can work in a complementary or synergistic way.

## Methods

Both feature generation events (i.e., mapathon and AFE) studied here used the same satellite imagery. The study area comprises two districts in Central Asia that were inaccessible to EI at the time of the study. To protect the security of populations living in our study region, the specific geographic areas will not be disclosed. The varied terrain of the urban and rural study areas included rocky and forested mountainous regions, low plateau areas with desert terrain, and some fertile plains used for farmland. The climate in the study area is arid to semiarid with low rainfall in most areas of the region. Permanent and temporary housing structures were visible in the satellite imagery used for the study and included small mud free-standing structures, larger mud-brick and stone compounds surrounded by walls, modern free-standing structures and housing complexes in urban areas, small cliff dwellings, and temporary tents or yurts.

### Mapathon

We conducted the mapathon for this project under the guidance of the WHO and the Geospatial Research, Analysis, and Services Program (GRASP) at the Centers for Disease Control and Prevention (CDC). The mapathon coordinators created a dedicated ArcGIS Online (ESRI, Redlands, CA) hub page for enrolling and training both novice and experienced mappers. This mapathon resource repository included registration information, tutorials on digitizing and application use, a real-time monitoring dashboard to analyze participant progress, links to communication channels, and sections on frequently asked questions. Mapathon participants from the CDC and WHO were recruited via emails and hardcopy informational posters. Participants logged into an ArcGIS Online web application with basic data editing functionality to view current, high-resolution (0.3–0.5 m) satellite imagery downloaded from DigitalGlobe (DG) with the goal of identifying structures inside the two study districts.

**Fig. 1:**
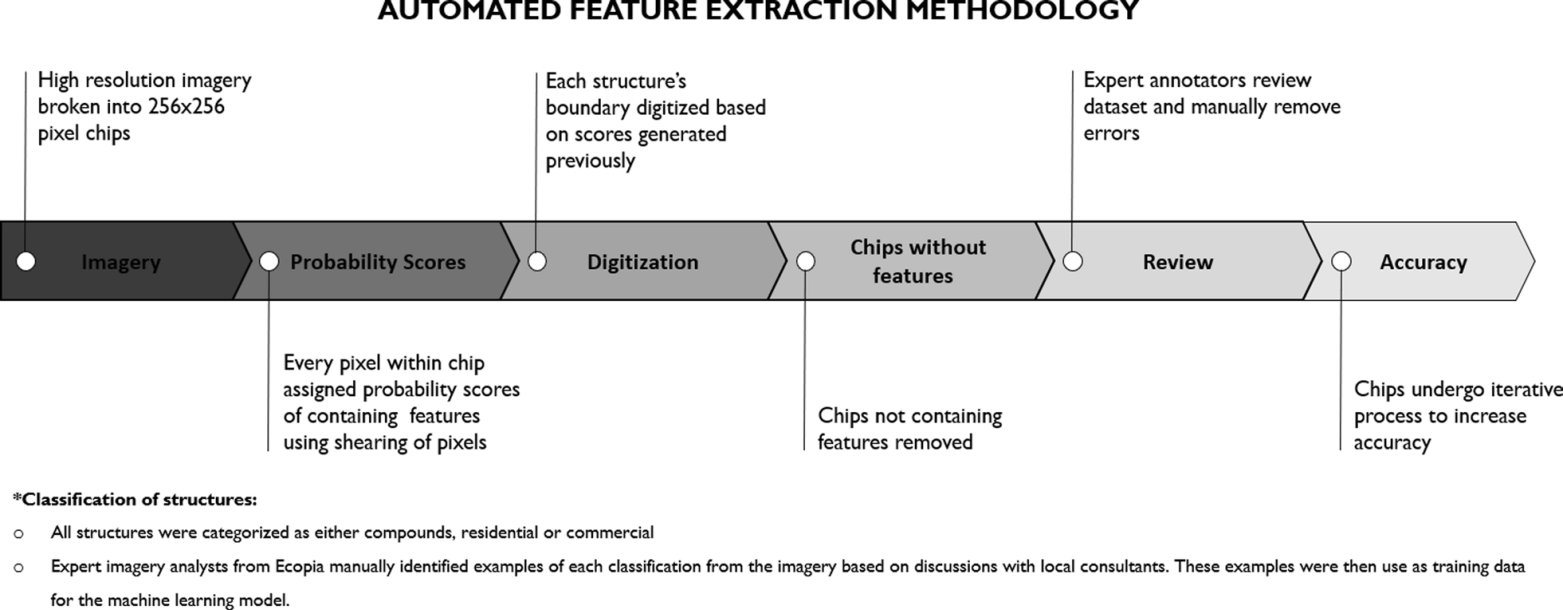
Feature generation using two methods: mapathon (point) and automated feature extraction algorithm (polygon)

The imagery for both districts covered a total area of 6146 square kilometers. The entire area of interest was divided into 1 km × 1 km grid cells for the contributors who then digitized features of interest within one cell at a time. For each cell, contributors placed one spatially linked point on the center of any man-made structure that was larger than 9 m^2^ in the image (Fig. 1). If multiple structures existed within a compound (i.e. several structures surrounded by a common wall), the contributors digitized any eligible structure within the compound rather than counting the compound grouping of structures as just one point. Digitized features could be structures used for any purpose. Additionally, contributors were instructed to place points on structures that seemed to be under construction, regardless of shape, while avoiding those that appeared to be destroyed. Structures partially within the grid were treated as within the grid and were digitized. When a contributor marked a cell as complete, all man-made structures larger than 9 m^2^ and visible in the imagery should have been digitized as point feature class data (a discrete location represented by longitude and latitude coordinates). GIS experts served as validators within the mapathon coordination team, using a separate ArcGIS Online web application to validate any cells marked as complete by the contributors. Validators did not evaluate the quality of digitized points submitted by each contributor but ensured that all features of interest in the underlying satellite image were correctly digitized by contributors and made edits as needed before finalizing each cell. Because the mapathon contributors and validators had little-to-no knowledge about the setting, they did not make any classifications regarding the current use of the buildings they digitized.

### Automated feature extraction

**Fig. 2:**
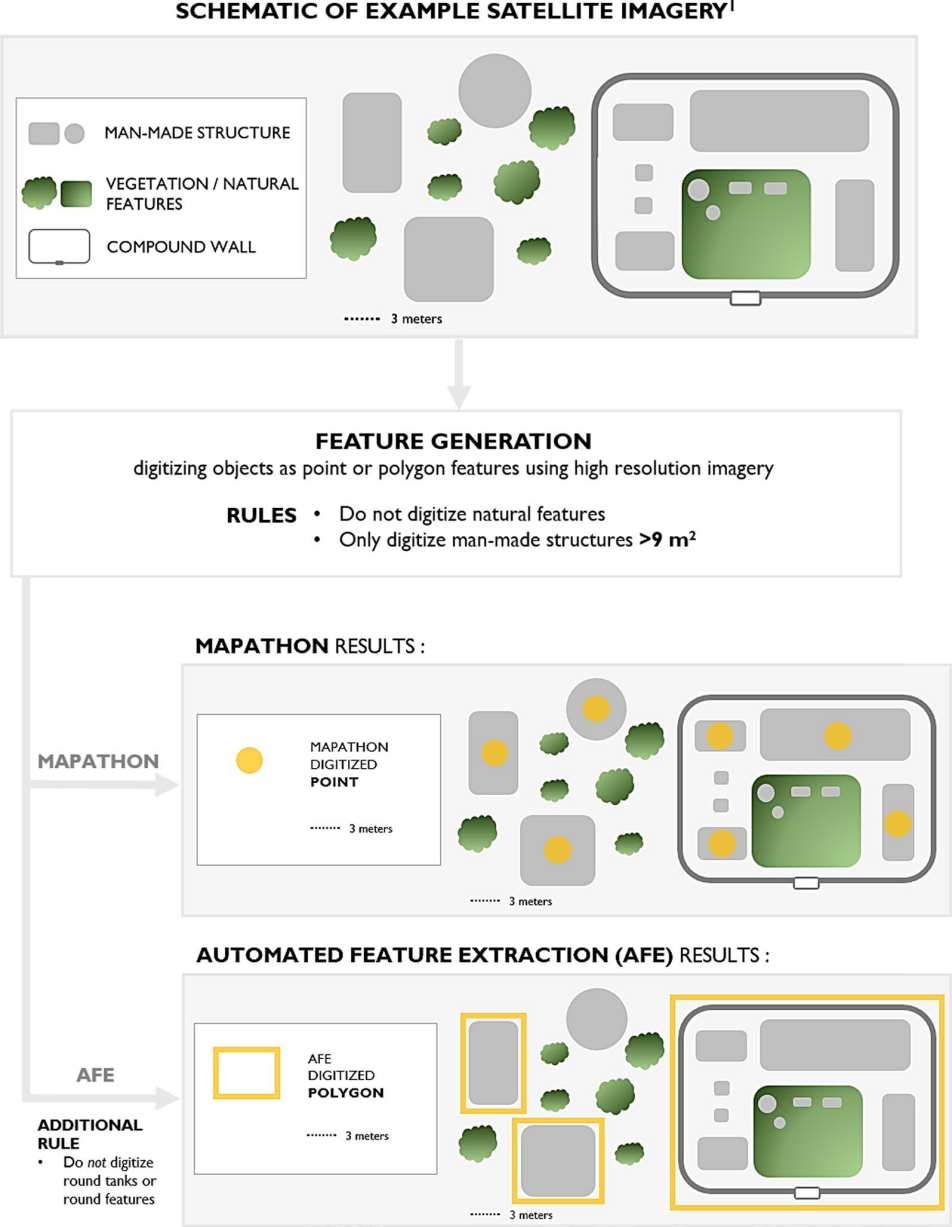
Sequence of steps employed for automated feature extraction (AFE)

The alternative method of acquiring spatial data for this project leveraged semi-automated feature extraction (Fig. 2) using the results from machine-learning deployments on millions of structures across various developing countries. The results gathered from previously conducted deployments supported Ecopia Tech’s (Toronto, ON, Canada) proprietary machine-learning models in generating building footprints for structures of interest detected in the imagery.

**Fig. 3:**
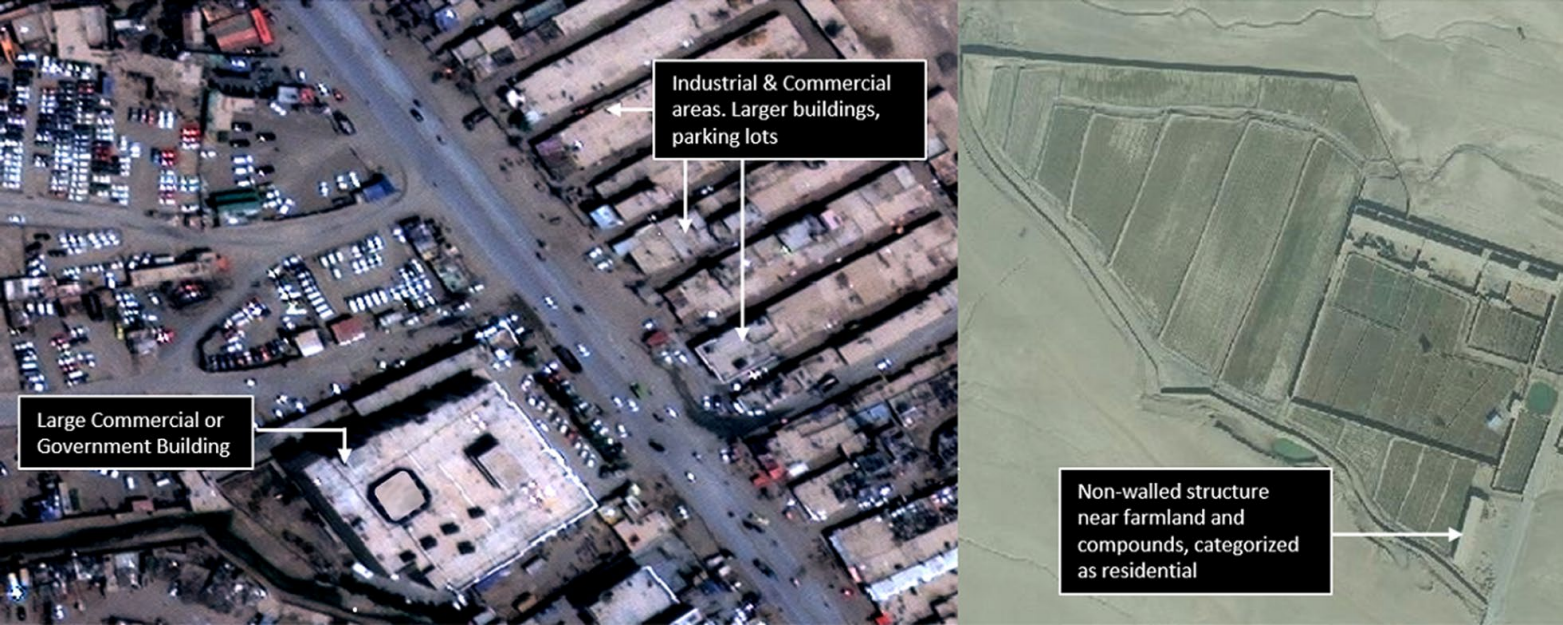
Example of free-standing structures in the study area, categorized as commercial (left) and residential (right) (© 2020 Maxar Technologies)

To support the models in extracting building footprints, relevant imagery was broken into a grid of 256 × 256-pixel chips. Within each chip, a classifier ran through every pixel and assigned each one a probability score for containing a feature of interest, using a variety of textural feature data from neighboring pixels in its calculations. The classifier algorithm used is a proprietary algorithm developed by Ecopia which measures the shearing of pixels along with color gradients to determine the likelihood that a structure falls within a pixel. Shearing in straight and/or circular lines can be indicative of man-made materials. If sheering, texture and contrast scores exceeded Ecopia’s internal threshold of 1 then the pixels were classified as likely containing or being a part of a structure. The classifier algorithm then digitized each structure’s boundary, using the confidence scores previously generated for each pixel. Any chips that did not contain structures were removed from the algorithm’s output. A team of former geospatial professionals and remote sensing enthusiasts who are expert annotators then reviewed the resulting data sets, manually corrected any errors, and provided any necessary updates to the classifier algorithm. Using a “CrowdRank” algorithm [32] we were able to classify users who perform better when compared against other users completing the same task. Users who regularly fall below a pre-defined benchmark are removed from the project in an iterative fashion to promote the highest accuracy possible. Informed by the updated and improved data, the classifier algorithm then iteratively reproduced the process to increase overall accuracy. During these iterations, the annotators continued to manually revise any incorrectly generated vector edges and updated the classifier algorithm accordingly. Furthermore, the annotators manually digitized obscured structures to reflect accurate footprints of structures of interest. Prior to this deployment, Ecopia Tech developed an AFE algorithm capable of classifying footprints and partnered with Maxar Technologies (Westminster, CO) to utilize their very high-resolution imagery mosaics and guidance on categorizing the building footprint outputs. To accurately categorize footprints as commercial, compound, or residential, expert imagery analysts from Ecopia manually identified examples of each from the imagery to use in training data for the machine-learning model. Guided by discussions with local consultants, Ecopia defined a compound as typically including several structures along with a yard surrounded by a wall. Non-walled, free-standing structures were then categorized as either commercial or residential depending on other contextual factors, such as the proximity and presence of latrines, farmlands, vehicles, and other indicators of human activity (Fig. 3). The AFE algorithm excluded structures that were round and smaller than 9 m^2^ to ensure boulders were not mistaken for structures. The outputs of the model were polygons drawn around the perimeter of all free-standing structures larger than 9 m^2^ (whether categorized as commercial or residential) and all residential compounds (Fig. 1). Unlike the mapathon, compounds, regardless of the number of structures contained inside, were treated as one polygon feature.

An estimate of the population inhabiting the structures captured by each method was calculated employing the assumption that a compound includes three households, where each household has an average of 2 adults and 5 children. Therefore, it was estimated that each compound housed an average of 21 individuals.

### Accuracy assessment

The study areas were selected for two reasons: their geographic heterogeneity despite the low spectral diversity (e.g., deserts, arid mountainous, alluvial plains, etc.) and the inaccessibility of local ground-truth data due to continuous insecurity.

We employed the following accuracy assessment techniques to determine how well each feature generation method—mapathon or AFE—captured the actual location of features of interest.

#### Assessment 1

We conducted an agreement analysis of the two feature classes to assess matches across the two outputs. To ensure a uniform comparison across both sets of features, we only considered the non-compound AFE features and the mapathon points that were not part of a compound. A simple ‘select by location’ query was used within ArcGIS Pro, whereby both feature types, point and polygon, were analyzed together.

**Fig. 4:**
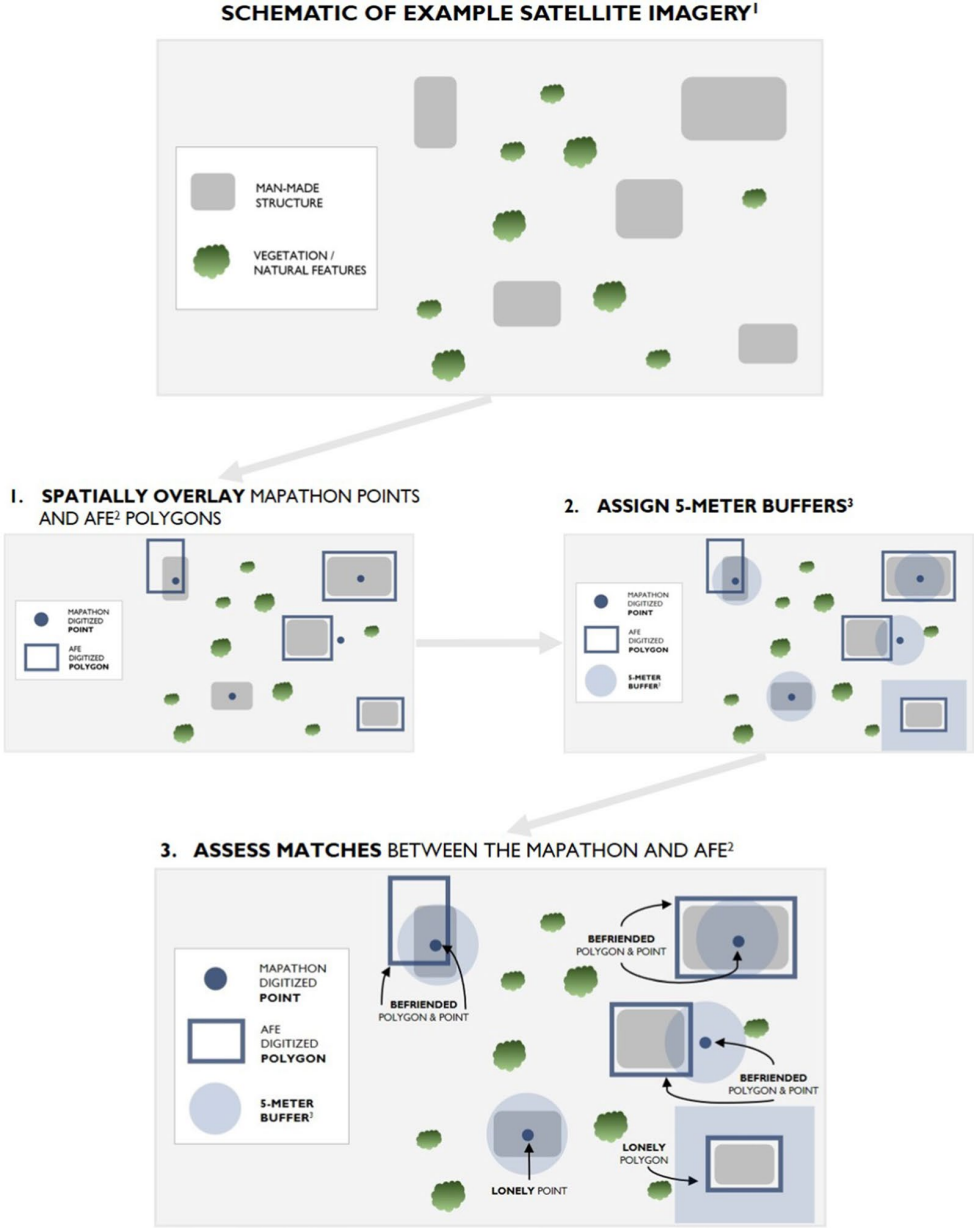
Comparing the results of two feature generation methods: match assessments categorized as “befriended” or “lonely”. ^**1**^For illustrative purposes only. ^2^AFE = automated feature extraction. ^3^Five-meter buffers were measured around each point and measured from the edges of each polygon. For clarity of illustration, 5-m polygon buffers are only shown for the lonely polygon

To allow for small shifts in geographic location when comparing mapathon points and AFE polygons, features within 5 m of another polygon’s perimeter were considered a match and labeled as “befriended” (Fig. 4). If a point from the mapathon did not fall within an AFE polygon or have an AFE polygon within 5 m of it, we labeled that point as “lonely”. Similarly, if a polygon from AFE did not have a corresponding mapathon point within 5 m of the polygon’s edge, we labeled that polygon as “lonely”. Five meter buffers were applied to points and polygons separately, rather than simultaneously, such that the consideration of a potential 5 m shift in the location of the polygon or the point was analyzed first for one of the feature types and then the other.

#### Assessment 2

We conducted a subset analysis of the data from both feature generation methods. Two GIS experts who were not part of the mapathon validation team, independently analyzed the same set of 100 random, lonely points and 100 random, lonely polygons against the same high-resolution imagery. The subset analysis was limited to lonely points and lonely polygons as lonely features were not considered to be matches from Assessment 1. The GIS experts did not have access to ground truth due to security reasons in the study region. As an alternative, high-resolution satellite imagery was used as the source of verification for their assessment. They classified points and polygons correctly corresponding to a structure as true positives (TP) based on verification against satellite imagery and classified the remaining features as false positives (FP), also based on verification against satellite imagery. Finally, the true positive percentage was calculated by averaging the number of TP and FP yielded by the two GIS experts.

#### Assessment 3

The third accuracy assessment involved statistically comparing the features generated from both the mapathon and AFE to a microplan, considered the gold-standard data, shared by the local-level team from one of the study districts. The microplan was developed by the local health authorities and is created by listing out the known settlements in the areas targeted for vaccination and estimating the number of households that vaccination teams should expect to find in each settlement. The study district consisted of 44 operational sub-districts called clusters and one vaccination team was assigned to work in each cluster. The microplan included cluster names, number of households per cluster, number of vaccination teams, the population aged 0–59 months (i.e., target age for vaccination), and total population.

To account for differences in feature extraction techniques and parameters, we analyzed residential or compound polygons and mapathon points. Because mapathon points captured structures of any use while the AFE and microplan indicated household structures of residential use only, the count of mapathon points per cluster was recalculated to better approximate the number of households in the cluster. To count only one mapathon point per compound, we first removed mapathon points that fell inside of AFE polygons categorized as compounds from the analysis. We then multiplied the percentage of AFE compounds containing at least one mapathon point (83%) with the number of compounds in each of the 44 clusters and added that number to the mapathon points for each cluster, thus creating a more comparable dataset to the AFE polygons and microplan.

The null hypothesis for this assessment assumed no significant differences between the average number of features per cluster in the microplan in comparison with the average number of features per cluster obtained from each feature generation method. To test this assumption, we conducted 2-sample t-tests: (a) comparing the average number of points per cluster from the mapathon to the microplan and (b) comparing the average number of polygons per cluster from AFE to the microplan. T-statistics indicated whether there were significant differences (p < 0.05) between each feature generation method and the microplan.

#### Assessment 4

Population in the study area was estimated by applying the following assumptions to the mapathon and AFE data—A compound consists of 3 households and a household consists of 7 individuals. This assumption was based on advice from in-country colleagues. Therefore, population estimates were calculated based on the number of free-standing (7 individuals) residences and the number of compound residences (21 individuals).

## Results

### Descriptive statistics of mapathon and validation

The mapathon took place in August 2018 over five days and recruited 107 participants. Seven organizers spent approximately 840 h, or approximately 120 h per person, preparing for and conducting the event. The contributors and validators captured a total of 92,713 valid individual structures across an area of 6146 km^2^ during the mapathon. The total number of individual structures did not take into account, the adjustments made by validators where mapathon points of insufficient quality were deleted. The number of digitized features differed widely between participants, with a minimum point count of 1 and a maximum of 10,134. Participants spent a total of 98 h digitizing, averaging 748 features per person.

Of the 92,713 structures digitized during the mapathon, the vast majority (n = 79,640, 85.9%) required no revision by a validator, a sizeable proportion (n = 12,608, 13.6%) were uniquely generated by the validators because contributors missed these structures entirely, and < 0.5% were edited by the validators or contributors themselves.

### Descriptive statistics of automated feature extraction

The AFE process required a total of nine days to complete, costing $25,000. This cost included image mosaic preparation, training data development, model deployment and iterations, and quality checks for a total area of 6146 km^2^. The semi-automated method identified 53,150 individual structures and compounds. The combined use of Maxar satellite imagery processing and Ecopia algorithms enabled the generation of building footprints and consequent categorization of those footprints. Due to the difference in methodologies, it is expected that the AFE method would result in fewer features than the mapathon. The AFE classifier categorized 80.7% (n) of the building footprints as compound structures, 16.4% (n) as commercial structures, and 2.9% (n) as residential structures. The average area of all AFE polygons, representing the footprints of compounds, was 808.2 m^2^, while the average area for commercial and residential building footprints were 24.4 m^2^ and 65.3 m^2^, respectively.

### Assessment 1: Comparing mapathon and AFE

Based on the matches assessed across the two feature generation outputs (Fig. 4), 30% (n/N) of the non-compound AFE-identified structures intersected or were within 5 m of a mapathon point, while 70% (n/N) were not. Comparatively, 28% (n/N) of the mapathon points that were not part of a compound fell inside of or were within 5 m of an AFE polygon, while 72% (n/N) did not. A slightly higher proportion of AFE features were befriended (30%) than the proportion of mapathon points that were befriended (28%). 2% more identified polygons were corroborated by a mapathon point than identified mapathon points were corroborated by a polygon.

### Assessment 2: Subset analysis

The subset analysis demonstrated that the AFE method, including residential and commercial structures, had a higher true positive percent (90.5%) than the mapathon (84.5%) in identifying structures (see Appendix 1).

### Assessment 3: Comparing average number of features per cluster against the microplan

**Fig. 5:**
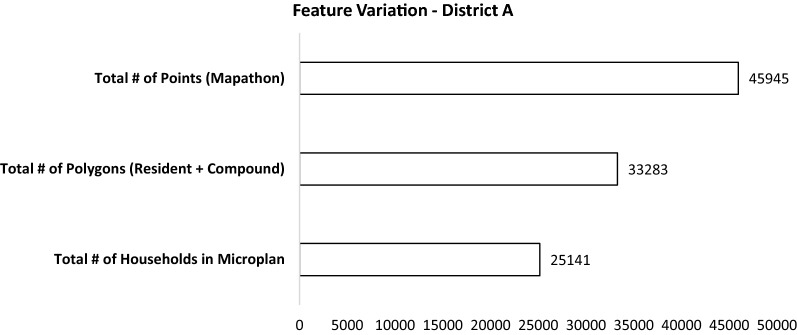
Count of features across all clusters by feature generation or listing technique

When compared against the 25,141 total features included in the microplan, the mapathon identified an additional 20,804 features. The difference between the microplan and the AFE results was smaller (8142). Figure 5 shows the variation of all features, resulting from the three different techniques in one district of the study area.

The average number of features per cluster in the microplan was 571.39 and the average number of mapathon points per cluster was statistically significantly higher (mean = 1044.20, t = 3.56, p < 0.001) as was the average number of AFE polygons per cluster (mean = 756.43, t = 2.09, p = 0.04). Both comparisons indicate that the microplans were missing structures in the clusters reviewed or that both the methods overestimated the number of structures in the microplan clusters. The p-value was significant (alpha = 0.05) for both t-tests, providing sufficient support to reject the null hypothesis, which assumed no significant difference between the average number of features obtained through both extraction methods and the microplan (Table 1).

### Assessment 4: Estimating population

The population in the study area estimated from mapathon results was 648,991 and the population based on AFE results was 911,302.

**Table 1:** Two-Sample t-Test results comparing features per cluster

t-Test: two-sample assuming unequal variances					
	Mapathon points	Microplan		AFE features	Microplan
Mean	1044.20	571.39	Mean	756.43	571.39
Variance	717,621.79	60,285.87	Variance	284,076.30	60,285.87
Observations	44	44	Observations	44	44
Hypothesized mean difference	0		Hypothesized mean difference	0	
df	50		df	60	
t Stat	3.56		t Stat	2.09	
P(T ≤ t) two-tail	0.00083		P(T ≤ t) two-tail	0.04071	
t Critical two-tail	2.01		t Critical two-tail	2.00

**Table 2:** Comparison of feature generation techniques

Mapathon	Indicator	Automated feature extraction
A. generic indicators		
60 days	Time	9 days
Cost of organizational ArcGIS Online licenses (creator license = $1000/year, Editor license = $200/year). Labor cost for coordinators (based on salary of coordinators). Participants were unpaid volunteers	Cost^a^ to conduct project	$25,000 for 6146 km^2^
Specialized application development expertise required of coordinators	Skill level	Specialized machine-learning expertise required
Smaller geographic regions	Area (best suited for)	Larger geographic areas
B. Performance Indicators		
92,713	Number of structures identified	53,150
+20,804	Difference in structures identified compared to microplan	+8142
28%	Non-compound match rate (%befriended)	30%
30,904	Number of compounds identified	43,395
84.50%	True positive percent	90.50%
648,991	Estimated population^b^	911,302

^a^Cost does not take cost of imagery into account, as imagery did not have a stand-alone procurement fee for the specific event studied here

^b^Population was estimated by applying the following assumptions to the mapathon and AFE data: a compound consists of 3 households and a household consists of 7 individuals

## Discussion

The results obtained from both feature generation methods were compared to estimates from the current field-level source, a microplan line-list. Even though the study compared differing methodologies for feature generation, measures to ensure a uniform comparison were taken into consideration, including the exclusion of commercial structures and recalculation of mapathon points to better approximate the number of households in the cluster. Results of the t-tests indicated statistically significant differences for each technique in comparison with the microplan, with the total number of features per cluster larger than the microplan and in the case of the mapathon, almost twice the average. The AFE was found to be similar to the microplan when looking at absolute feature counts. While the accuracy of the field-level microplan itself is unknown, it is the best comparison dataset the authors had to compare mapathon and AFE results to. AFE outputs had a higher true positive percent (90.5%) than the mapathon (84.5%), meaning the AFE was slightly better at correctly identifying a structure in the satellite imagery as a structure. The two techniques could be optimized to more accurately detect structures, as both were subject to false positives and an unknown number of false negatives. Large boulders and trees were accidentally digitized manually as structures in the mapathon which could be avoided by using various indices that enhance spectral diversity and by conducting more training with the participants. Likewise, numerous structures amidst cliffs and hilly terrain were not captured by the AFE technique.

Population estimates in inaccessible regions are often difficult to ascertain due to dynamic population changes and the enumeration process being labor-intensive [12]. This has important consequences for planning immunization campaigns and estimating vaccination coverage for EI. Acquiring precise population estimates translates into improved vaccine delivery programs once areas become accessible and more accurate evaluations on the coverage of the campaign [33]. This analysis was able to produce rough population estimates for the study area derived from each feature generation method, based on structure to population ratio assumptions supplied by country level partners. Our overarching purpose in comparing both feature generation methods was to determine which method more accurately identified structures in high-resolution satellite imagery and how the two methods might best complement one another. The most accurate population estimates are a result of optimum accuracy in structure identification.

As populations and population movements continue to fluctuate across large geographic areas, the availability of up-to-date information on the distribution of human settlements constantly changes [34]. These are circumstances in which AFE that can be rerun and retrained quickly could make valuable contributions to data availability compared to mapathons that require time-consuming manual inspections for updates. This AFE process required two days for mosaic preparation, and seven days for training data curation, model deployment iterations, and quality checks. Algorithms like the ones used here are useful for expediting work while maintaining or enhancing quality; however, these algorithms are costly. The cost of the pilot AFE project was $25,000 across 6146 km^2^ of the study area and required highly specialized technical expertise (Table 2). As this technology becomes more commonly used and explored, it is expected that the cost could decrease over time, making it more accessible. In contrast, participatory and collaborative mapping like mapathons require an extensive amount of manpower and time, making it much harder to translate into monetary costs and are therefore most valuable when timeliness is less of a priority, the geographic scale of the study is limited, and current, high-resolution imagery is available (Table 2).

The use of mapathons for public health interventions has increased meaningfully in recent years [35]. Mapathons have the ability to promote effective community engagement, creating a sustainable mechanism of generating geographic data that can be used by local immunizers during campaigns, ensuring the inclusion of all settlements. This collaborative style of mapping can recruit a range of expertise and be conducted mostly free of cost; however, the two main methodological challenges are the uncertainty of the quality of data generated by participants and the number of hours it takes to organize and conduct a mapathon.

Manual feature generation and model-driven feature generations are also useful methods to utilize in tandem to exploit the merits of each and develop a product superior to that which would be created by using only one method alone. For example, smaller scale mapathon efforts are an efficient means of training data creation for AFE. Additionally, AFE footprints can be added to an online application to assist mapathon validators in assessing incoming results during a mapathon event. Both AFE footprints and mapathon points, or a combination of the two, can be utilized as inputs for population estimation models whereby structures are a proxy for the population. The researchers suggest that parameters, such as terrain type, delivery deadline, budget, human resources, computing resources, imagery availability, and requirements of the data output should be considered to strike an appropriate balance in using these two methods together or to decide if one method is more favorable than the other for a particular project. Although assessing whether the mapathons together with AFE provide more accurate results was outside of the scope of this paper, future work might include an analysis of how these two methods could be used in tandem.

## Limitations

While the strengths of this study included the use of current, equivalent satellite imagery across the two feature generation methods compared, multiple assessments to understand the ways in which the results of each method were similar and different, and a thorough logistical comparison on how to decide which method to employ (when you must choose only one), the study also had limitations. An important caveat for interpreting our findings is the lack of a true gold standard, in the form of ground-truthed data collection, which made it impossible to calculate the false-negative rate for each method. Furthermore, due to the insecurity of the area, building footprints and points generated through the study could not be validated in the field for potential inaccuracies. Instead, the findings were compared to a microplan line-list developed by the country’s local teams and considered closest to ground truth. However, microplans are also limited because they capture known areas of settlements and may not reflect newly established or abandoned settlements. Finally, the over and underestimation of structures extracted through both techniques cannot be investigated on ground due to pending security access within the region. The authors suggest replicating this study in accessible areas to evaluate and compare findings. Another inherent challenge in this study is the use of different feature extraction techniques. The mapathon participants were instructed to place one point per unique rooftop included within compounds, while the AFE grouped numerous structures into one feature (as shown in Fig. 1) when the polygon feature represented a compound. This resulted in a considerable underestimation of individual structures (39,563 fewer structures) with the AFE technique, which can be an issue if using individual structure counts to estimate population.

Another important limitation is the lack of equivalent, constant oversight and the introduction of human bias by the mapathon participants in comparison with AFE. While mapathon participants were provided with training resources and had access to constant communication with GIS experts through an online chat application, mapathon coordinators were not able to monitor every point placed by novice contributors. Mapathon validators did confirm the accuracy of each digitized point and made any necessary edits following submission by the contributors, but human error could still be present in this validation process. The AFE method involved in this study also utilized human input as part of the validation process after the classification algorithm was run, but the possibility of human error is lower than that of the mapathon because much less human input was involved.

## Conclusions

We presented results comparing two feature extraction methods with the objective of determining how accurately each method identified settlements in hard-to-reach areas for the purpose of improving EI efforts. The findings suggest that the AFE is more robust in detecting structures when compared to the mapathon; however, the results need to be validated in the field when feasible in order to calculate sensitivity and specificity compared to the gold standard of data collected on the ground (i.e., ground truthing). AFE could be particularly useful for essential immunization efforts because it generates spatial data from imagery rapidly and has the potential to be more accurate than mapathons. The geographic data obtained from this study will be used to improve existing microplans with the intent of increasing the EI coverage rate in our study area. Future comparison studies must consider a consistent methodological framework across both feature extraction techniques to improve the findings presented in this study. Although both feature generation techniques could be improved further, this study is a step towards strengthening the understanding of potential methods of mapping population distribution in inaccessible areas to support public health interventions.

## Data Availability

The datasets generated during and/or analyzed during the current study are not publicly available to protect the security of populations living in our study region but are available from the corresponding author on reasonable request.

## Appendix 1

Assessment 2: Subset analysis results.

**Table:**

GIS Analyst	Mapathon points		AFE polygons	
	True positive	False positive	True positive	False positive
A	91	9	94	9
B	78	22	87	13
Total	169	31	181	22
Average percent	84.5%	15.5%	90.5%	11%

## Abbreviations

AFE: Automatic feature extraction
CDC: Centers for Disease Control and Prevention
DG: DigitalGlobe
EI: Essential immunization
FP: False positives
GIS: Geographic information system
GRASP: Geospatial Research, Analysis, and Services Program
RED: Reach every district
TP: True positives
UNICEF: United Nations Children’s Fund
VHR: Very high-resolution
WHO: World Health Organization
